# Identifying cellular markers of focal cortical dysplasia type II with cell-type deconvolution and single-cell signatures

**DOI:** 10.1038/s41598-023-40240-3

**Published:** 2023-08-16

**Authors:** Isabella C. Galvão, Ludmyla Kandratavicius, Lauana A. Messias, Maria C. P. Athié, Guilherme R. Assis-Mendonça, Marina K. M. Alvim, Enrico Ghizoni, Helder Tedeschi, Clarissa L. Yasuda, Fernando Cendes, André S. Vieira, Fabio Rogerio, Iscia Lopes-Cendes, Diogo F. T. Veiga

**Affiliations:** 1 Department of Translational Medicine, School of Medical Sciences, University of Campinas (UNICAMP), Campinas, Brazil; 2 Department of Pathology, School of Medical Sciences, University of Campinas (UNICAMP), Campinas, Brazil; 3 Department of Neurology, School of Medical Sciences, University of Campinas (UNICAMP), Campinas, SP Brazil; 4 Department of Structural and Functional Biology, Institute of Biology, University of Campinas (UNICAMP), Campinas, Brazil; 5 The Brazilian Institute of Neuroscience and Neurotechnology (BRAINN), Campinas, Brazil

**Keywords:** Computational biology and bioinformatics, Neurology, Neurological disorders

## Abstract

Focal cortical dysplasia (FCD) is a brain malformation that causes medically refractory epilepsy. FCD is classified into three categories based on structural and cellular abnormalities, with FCD type II being the most common and characterized by disrupted organization of the cortex and abnormal neuronal development. In this study, we employed cell-type deconvolution and single-cell signatures to analyze bulk RNA-seq from multiple transcriptomic studies, aiming to characterize the cellular composition of brain lesions in patients with FCD IIa and IIb subtypes. Our deconvolution analyses revealed specific cellular changes in FCD IIb, including neuronal loss and an increase in reactive astrocytes (astrogliosis) when compared to FCD IIa. Astrogliosis in FCD IIb was further supported by a gene signature analysis and histologically confirmed by glial fibrillary acidic protein (GFAP) immunostaining. Overall, our findings demonstrate that FCD II subtypes exhibit differential neuronal and glial compositions, with astrogliosis emerging as a hallmark of FCD IIb. These observations, validated in independent patient cohorts and confirmed using immunohistochemistry, offer novel insights into the involvement of glial cells in FCD type II pathophysiology and may contribute to the development of targeted therapies for this condition.

## Introduction

Focal cortical dysplasia (FCD) is a malformation characterized by structural and cellular abnormalities of the cerebral cortex, such as disrupted organization of the neuronal layers and abnormal cell development. The condition is often difficult to treat with available anti-seizure medication and may require surgical resection of the affected brain tissue. FCD is a major cause of epilepsy in children, accounting for up to 50% of epilepsy surgeries in this age group^1^. FCDs are classified into three categories, each with its distinct histopathology and genetic alterations^2,3^. Among them, FCD type II is the most common^3^ and is distinguished from other types of FCDs due to loss of cortical organization in addition to the presence of dysmorphic neurons and balloon cells, which are mixed lineage immature cellular entities expressing both neuronal and glial proteins. At the histopathology level, FCD II can be further sub-classified into FCD IIa and IIb, presenting with dysmorphic neurons only (IIa) or with both dysmorphic neurons and balloon cells (IIb). Some patients with FCD II harbor somatic mutations in the mTOR pathway, mostly in the FCD IIb subtype^3^.

Cell type deconvolution methods applied to gene expression data have been used to estimate the proportions of different cell types in heterogeneous tissues in both healthy and disease settings^4,5^. To infer cell type abundances, these methods use regression on a reference expression matrix containing a set of marker genes constructed from reference expression datasets, such as bulk RNA-seq of individual cell types. More recent deconvolution approaches have been adapted to use single-cell RNA sequencing (scRNA-seq) reference signatures^6–8^, which might help to detect rarer cell types in mixture samples.

The brain is a complex organ composed of several cell types including neurons, glial cells (astrocytes, oligodendrocytes, ependyma, and microglia), and endothelial cells. Previous studies assessing cell-type deconvolution specifically in brain tissues found that cell-type estimates obtained through deconvolution highly correlated with those obtained through experimental approaches such as single-nuclei RNA-seq^9^ and immunohistochemistry^10^. Thus, deconvolution has been shown to predict cell-type proportions in brain tissues accurately and can be useful for exploring the contribution of cell types in diseases such as FCD.

Due to their distinct histopathological and genetic features, FCD II subtypes are likely to have different cellular compositions. In this study, we aimed to compare the cellular landscapes of FCD IIa and IIb subtypes, which have not been systematically investigated using unbiased bioinformatic approaches, to understand similarities and differences in the tissue composition of these common types of focal epilepsies. To address this, we leverage previous transcriptomic studies along with cell-type deconvolution based on single-cell signatures to systematically characterize the cellular heterogeneity in tissue samples from patients with FCD IIa or IIb. We then applied immunochemistry techniques to validate our findings.

## Materials and methods

### RNA-seq data acquisition

FCD type II RNA-seq from Kobow et al.^11^ was retrieved from the European Nucleotide Archive (accession code SRP188422) and downloaded using the enaBrowserTools v. 1.1.0 (https://github.com/enasequence/enaBrowserTools). Raw FASTQ RNA-seq from Assis-Mendonça et al.^12^ was obtained from the authors.

### RNA-seq data processing

FASTQ files were processed using the RNA-seq nf-core pipeline v. 3.8.1^13,14^. Briefly, raw reads were trimmed using TrimGalore and aligned to the human reference genome hg38 using STAR^15^, followed by gene-level quantification using the RSEM^16^ tool based on the Gencode v. 40 human reference transcriptome. Only samples containing at least 15 million mapped reads with an RSEM mapping rate of at least 80% were further selected for downstream analyses. After quality filtering, the first dataset included 11 samples from Kobow et al.^11^ and 8 samples from Assis-Mendonça et al.^12^. To integrate these two independent RNA-seq datasets, we proceed as follows: gene-level counts were normalized using the variance stabilizing transformation (*vst*) from DESeq2^17^, and the function removeBatchEffect from the limma R package^18^ was applied to remove batch effects between the independent patient cohorts. For the second dataset (Zimmer et al.^19^), we used pre-processed RNA-seq of 32 patients obtained from the authors since raw FASTQ files could not be obtained from the original publication. The available clinical information for all samples is provided in Supplementary File 1.

### Cell-type deconvolution using CIBERSORTx

Given the mixture RNA-seq and cell-type signatures, CIBERSORT (Cell-type Identification By Estimating Relative Subsets Of RNA Transcripts)^4^ applies a machine learning approach known as ν-support vector regression (ν-SVR) to infer the fraction of cell types in the mixture. CIBERSORTx requires two input matrices for deconvolution: (i) a signature matrix comprised of marker genes learned from purified cell populations or single-cell RNA-seq, and (ii) a gene expression matrix from the bulk RNA-seq sample to be deconvoluted. More recently, CIBERSORTx^6^ was developed to allow the use of single-cell RNA-seq and inference of cell-type expression. Specifically, we applied the module “Impute cell fractions” of the CIBERSORTx tool with the following parameters: relative mode, S-mode batch correction, and disabled quantile normalization. Also, we tested four distinct reference signatures—named CA, VL, NG, and LK after the original studies—to perform FCD deconvolution. These signatures, containing the expression marker genes of major cell types in the brain, were obtained from Sutton et al.^9^ and were derived from single-nuclei RNA-seq data obtained from the human cerebral cortex. Signature matrices used to perform deconvolution are included in Supplementary File 2. The website version of the tool available at https://cibersortx.stanford.edu was applied to deconvolve FCD transcriptomes using the CA, VL, NG, and LK signatures. We also applied CIBERSORTx using the snRNA-seq dataset from Schirmer et al.^20^ (termed here SR signature), which profiled 48,920 individual nuclei from multiple sclerosis lesions and adjacent healthy tissue resected from the cortex. The local version of CIBERSORTx was applied to deconvolve FCD transcriptomes using the SR signature in a high-performance computer with 128G RAM and 16 processors. All plots were created in R v.4.1.2.

### Methylome-based deconvolution using EpiSCORE

We applied the EpiSCORE tool^21^ with default parameters to a set of FCD methylomes profiled using Methyl-seq^11^ using a brain-specific DNAm signature containing the promoter-level methylation of 113 cell-type-specific marker genes^22^. The mixture input matrix was created by obtaining the average gene-level methylation from Methyl-seq peaks located at the proximal promoter (1 kb upstream) of the transcription start site.

### FCD tissue collection

We collected specimens from 8 patients with epilepsy who underwent surgery for intractable seizures at the Epilepsy Surgery Program at Hospital de Clínicas, University of Campinas. All of the patients had a neuropathological diagnosis of FCD IIa (n = 4) or FCD IIb (n = 4), according to relevant guidelines and regulations, including the recommendations on immunohistochemical analysis presented by the International League Against Epilepsy^3^. All procedures were approved by the University of Campinas's Research Ethics Board, and written informed consent was obtained from all participants.

### Immunohistochemical analyses

Brain specimens were transversely cut oriented to the pia mater. Then, the tissues were fixed in buffered formalin (Sigma, St Louis, MO, USA). After 48–96 h, specimens were paraffin-embedded for immunohistochemistry (IHC). IHC was performed with primary antibodies against the glial fibrillary acidic protein (GFAP), an astrocytic marker (1:100, clone 6F2, Dako/Agilent, cat#M0761, Santa Clara, CA, USA), and MAP2, a protein expressed by neurons (1:1000, clone M13, Thermo Fisher, cat#13–1500, Waltham, MA, USA). Antibodies specificity was verified and IHC was performed as described in Mota et al.^23^ and Assis-Mendonça et al.^12^. Briefly, paraffin-embedded sections (4 µm) were incubated with each antibody (overnight; at room temperature). Afterward, a solution with the secondary antibody and peroxidase (AdvanceTMHRP^®^, Dako, cat#K4068, Glostrup, Denmark; or EnvisionTM Flex+, Dako, cat#K8002, Glostrup, Denmark) was added for 30 min at 37 °C. 3,3-diaminobenzidine was used as a chromogenic substrate and the sections were counterstained with hematoxylin. Control sections were concurrently submitted to the same protocol except for the primary antibody.

### Quantitative analysis of IHC

For quantitative analysis of immunoreactivity, a CS2 Aperio ScanScope scanner (Aperio Technologies, Vista, CA, USA) was used to obtain digitized images. For each individual, ten representative fields (20 × digital magnification) of the cortex immunoreacted for each marker (GFAP and MAP2) were evaluated. All digitized images were obtained under the same luminance and were further analyzed with ImageJ software, following the same criteria: (i) deconvolution of each image (separation of hematoxylin and DAB channels) was performed by using the IHC Profiler plugin^24^; (ii) the DAB channel was used for quantification with the threshold tool; (iii) for each image, a threshold value was established so that the histological findings were preserved in the most accurate way considering the original photo, that is, to exclude the low-intensity gray value of background staining and to allow the quantification of the positive-stained area detected in the soma and branches^12,25^. Groups were compared using the Wilcox rank-sum test in R.

## Results

### Cell-type deconvolution uncovers cellular alterations in FCD IIa and IIb lesions

**Figure 1:**
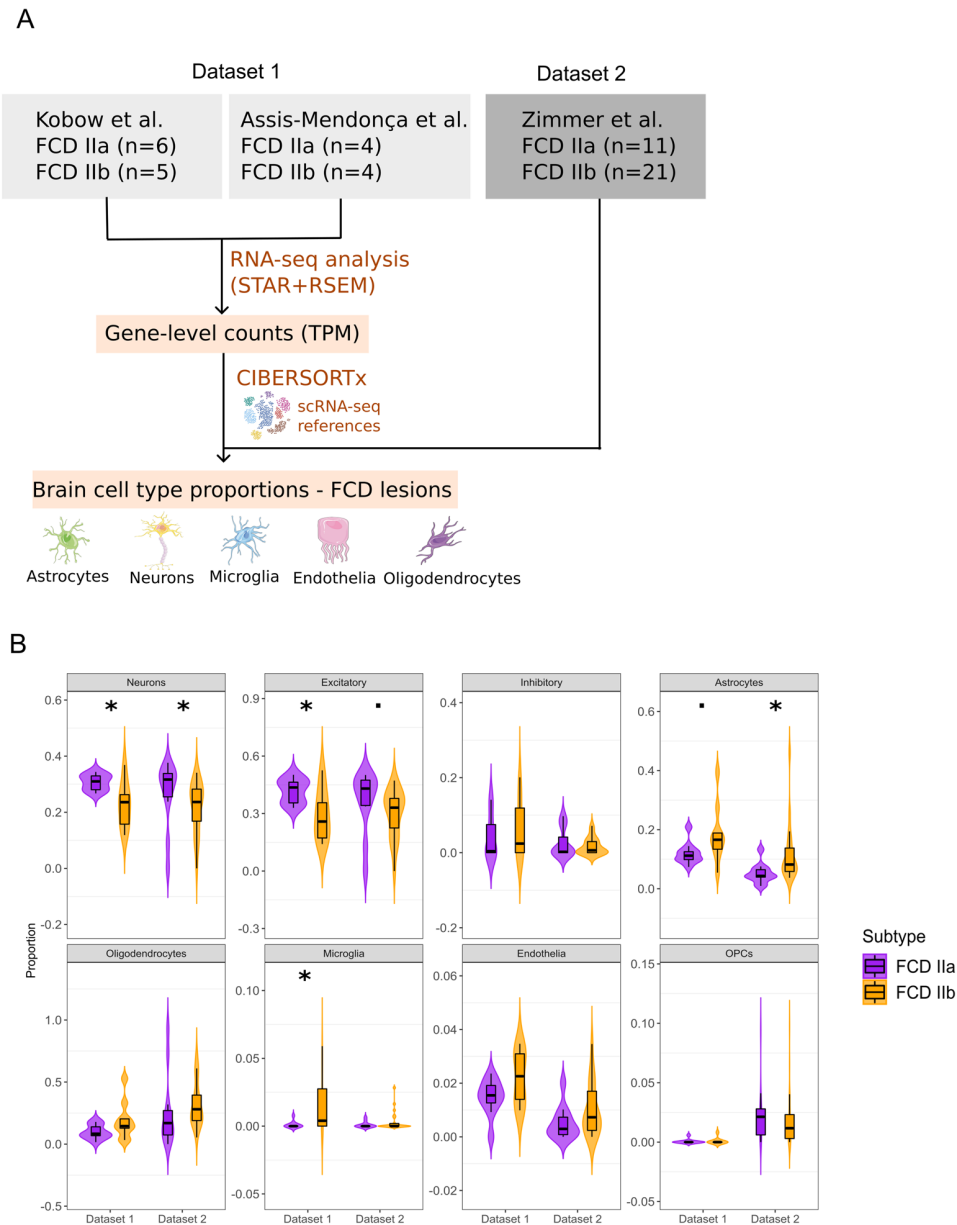
Cell-type deconvolution of major brain populations in FCD IIa and IIb lesions. (**A**) Schematic depiction of the analytical pipeline used for cellular deconvolution. RNA-seq datasets were obtained from three independent studies and were grouped into Datasets 1 and 2 (the number of samples is indicated). After RNA-seq data processing (see “Methods”), CIBERSORTx was performed to estimate cell type abundance using multiple single-cell reference signatures derived from snRNA-seq datasets of the human brain (see Fig. S1A). The Figure was partly generated using Servier Medical Art, provided by Servier, licensed under a Creative Commons Attribution 3.0 unported license. (**B**) Violin plot of cell type proportions in FCD IIa and IIb lesions in patients from Dataset 1 and Dataset 2, using the CA reference signature. The *y*-axis indicates the absolute cell type proportion (0 to 1) estimated by CIBERSORTx, while the *x*-axis indicates the patient dataset. The width of the violin indicates sample density, with the top, middle, and bottom of the black boxplot marking the 75th, 50th, and 25th percentiles, respectively. Significant changes between cell type estimates in FCD IIa and IIb lesions were detected using the Wilcoxon rank-sum test. ˙*p* < 0.1, *p < 0.5, **p < 0.01, ***p < 10^–3^, ****p < 10^−4^.

To estimate cell type proportions in patients with FCD IIa and IIb, we obtained bulk RNA-seq data from three independent transcriptomic studies and applied CIBERSORTx to obtain estimates of the brain-specific populations (Fig. 1A). To perform a robust analysis and ensure the reproducibility of findings regarding cellular changes between FCD II subtypes, we created independent discovery and validation cohorts with similar sample sizes. The discovery cohort (Dataset 1) included 10 FCD IIa and 9 FCD IIb samples, obtained after combining data provided by Kobow et al.^11^ and Assis–Mendonça et al.^12^. Before applying CIBERSORTx, RNA-seq was uniformly re-processed to obtain a gene expression matrix (see “Methods”), followed by normalization and batch effect removal to integrate these independent RNA-seq datasets (see “Methods”). After data integration, RNA-seq samples clustered by FCD subtype (Fig. S1), showing that FCD IIa and IIb lesions have distinct transcriptomic profiles and confirming that the data integration removed unwanted batch effects between datasets. The second dataset served as an independent validation cohort and included normalized gene-level expression data of 11 FCD IIa and 21 FCD IIb patients profiled by RNA-seq, obtained from Zimmer et al.^19^.

The cell-type signatures used in this study were derived from four studies that applied single-cell sequencing to profile the human brain (Fig. S2A): Hodge et al.^26^ (Cell Atlas or “CA” signature), Lake et al.^27^ (“LK” signature), Nagy et al.^28^ (“NG” signature), and Velmeshev et al.^29^ (“VL” signature). They include cell-type markers of major brain cell types, such as astrocytes, endothelia, neurons, excitatory neurons, inhibitory neurons, microglia, oligodendrocytes, and oligodendrocyte progenitors (OPCs). We chose these signatures based on a comprehensive benchmarking study^9^ comparing various single-cell signatures, which has found that the CA, NG, LK, and VL signatures had the best overall performance in deconvolving cell types in brain transcriptomes. When applied to deconvolution of FCD transcriptomes, the CA signature showed the best fit in both patient groups, as demonstrated by the Pearson correlation between the actual RNA-seq gene expression and the reconstructed gene expression by CIBERSORTx (Fig. S2B).

In the first patient cohort (Dataset 1), CIBERSORTx found a significant decrease in neurons, and expansion of astrocytes and microglia populations in FCD IIb lesions when compared to FCD IIa (Fig. 1B). The neuronal decrease was specific to the excitatory subtype, with no changes to inhibitory neurons (Fig. 1B). The analysis of the independent patient cohort (Dataset 2) confirmed the estimated loss of excitatory neurons and higher astrocyte abundance in FCD IIb (Fig. 1B). In addition, the decrease of excitatory neurons and increase of astrocytes was also detected by CIBERSORTx with other single-cell signatures VL, NG, and LK (Fig. S2C–E).

**Figure 2:**
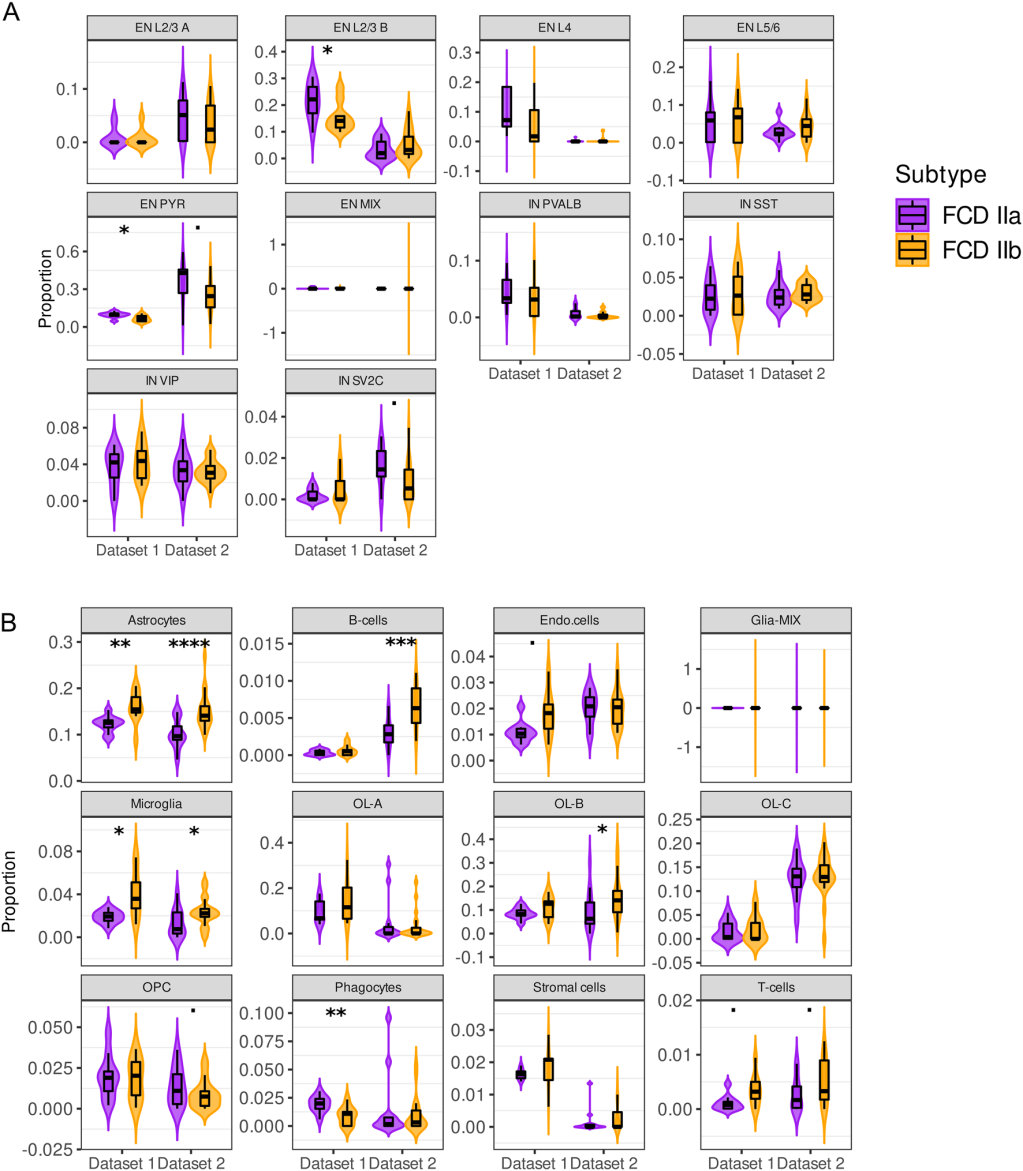
Cellular deconvolution of neuronal and non-neuronal subtypes in FCD IIa and IIb using the Schirmer (SR) signature. (**A**,**B**) Violin plots of estimated cell type proportions in FCD IIa and IIb lesions of (**A**) excitatory and inhibitory neurons, and (**B**) non-neuronal cells. The *y*-axis indicates the absolute proportion (0 to 1) estimated by CIBERSORTx, while the *x*-axis indicates the patient dataset. Significant changes between cell type estimates in FCD IIa and IIb lesions were detected using the Wilcoxon rank-sum test. ˙*p* < 0.1, *p < 0.5, **p < 0.01, ***p < 10^−3^, ****p < 10^−4^. EN = excitatory neurons, IN = inhibitory neurons, and PYR = pyramidal neurons. L indicates the cortical layer (e.g. L2/3 refers to layers 2–3), Endo = endothelia, OL = oligodendrocytes, OPC = oligodendrocytes progenitor cells, A/B/C = subclusters A, B, C.

Next, we wanted to explore which subtypes of excitatory neurons are affected in FCD IIb lesions. To address this question, we used CIBERSORTx and a snRNA-seq dataset from Schirmer et al.^20^, which represented a comprehensive single-cell survey of the brain cortex containing 18 neuronal and non-neuronal cell types. The Schirmer (SR) signature expanded upon the previous analysis by classifying excitatory and inhibitory neurons into subtypes across cortical layers and included a variety of glial and immune cells. The analysis found a specific decrease in excitatory neurons in the upper cortical layers 2/3 (cohort 1) and pyramidal subtypes (both patient cohorts), as shown in Fig. 2A. Microglia was significantly higher in FCD in both patient cohorts, which was also found with the previous deconvolution using the CA signature in patient cohort 1 (Fig. 1B). Additionally, the deconvolution with the SR signature confirmed an increase in the astrocyte compartment in FCD IIb lesions in both patient groups (Fig. 2B), consistent with the previous analyses using other signatures (Figs. 1B, S2C–E).

Finally, we compared FCD IIa and IIb cell-type frequencies with age-matched control samples obtained from the BrainSpan project^30^. These control RNA-seq samples were obtained from the frontal cortex of 8 individuals aged 1–40 years, which is in the same range as the FCD patients in this study. As expected, there were significant changes in several brain populations (Fig. S3), confirming that FCD type II lesions have overall a very distinct cellular composition from the healthy cortex. For instance, the analysis found significant microglia and astrocyte expansion in both FCD IIa and IIb subtypes. Yet, the increase of microglia and astrocyte subpopulations were more pronounced in the IIb subtype.

### Cellular alterations in FCD IIa and IIb inferred by methylome-based deconvolution

We also performed methylome-based deconvolution on a dataset of 6 FCD IIa and 5 FCD IIb samples profiled by Mehtyl-seq^11^ using the EpiSCORE tool^21^. This tool uses differentially methylated promoters to identify a set of cell-type marker genes (differentially expressed and specific to the cell type) in order to define tissue-specific signatures. Using this approach, Zhu et al.^22^ proposed and validated brain-specific DNA methylation (DNAm) signature containing the promoter-level methylation of 113 cell type-specific marker genes present in brain cell types.

We found that unsupervised principal component analysis (PCA) using DNAm can distinguish the FCD IIa and IIb subtypes, indicating their distinct methylation profiles (Fig. S4A). In addition, EpiSCORE estimated a decrease in neuron proportions and an increase in astrocytes in FCD IIb when compared to FCD IIa lesions (Fig. S4B). Thus, these results were consistent with the findings from transcriptome-based deconvolution with CIBERSORTx.

### FCD IIb lesions are characterized by enhanced reactive astrogliosis

The above cell type deconvolution showed astrocyte expansion in FCD IIb. To further investigate the role of astrocytes in FCD, we evaluated astrogliosis in FCD II subtypes using a gene signature containing 18 biomarkers associated with astrocyte activation in response to deleterious stimuli associated with neurological conditions^31^. This astrogliosis gene signature included cytoskeleton genes GFAP, vimentin (VIM), and nestin (NES), which are often upregulated in reactive astrocytes. It also included genes involved in cell signaling, such as the transcription factor STAT3, the Ca^2+^ binding transporter S100B, and secreted proteins such as the C3 complement factor and SERPINA3.

**Figure 3:**
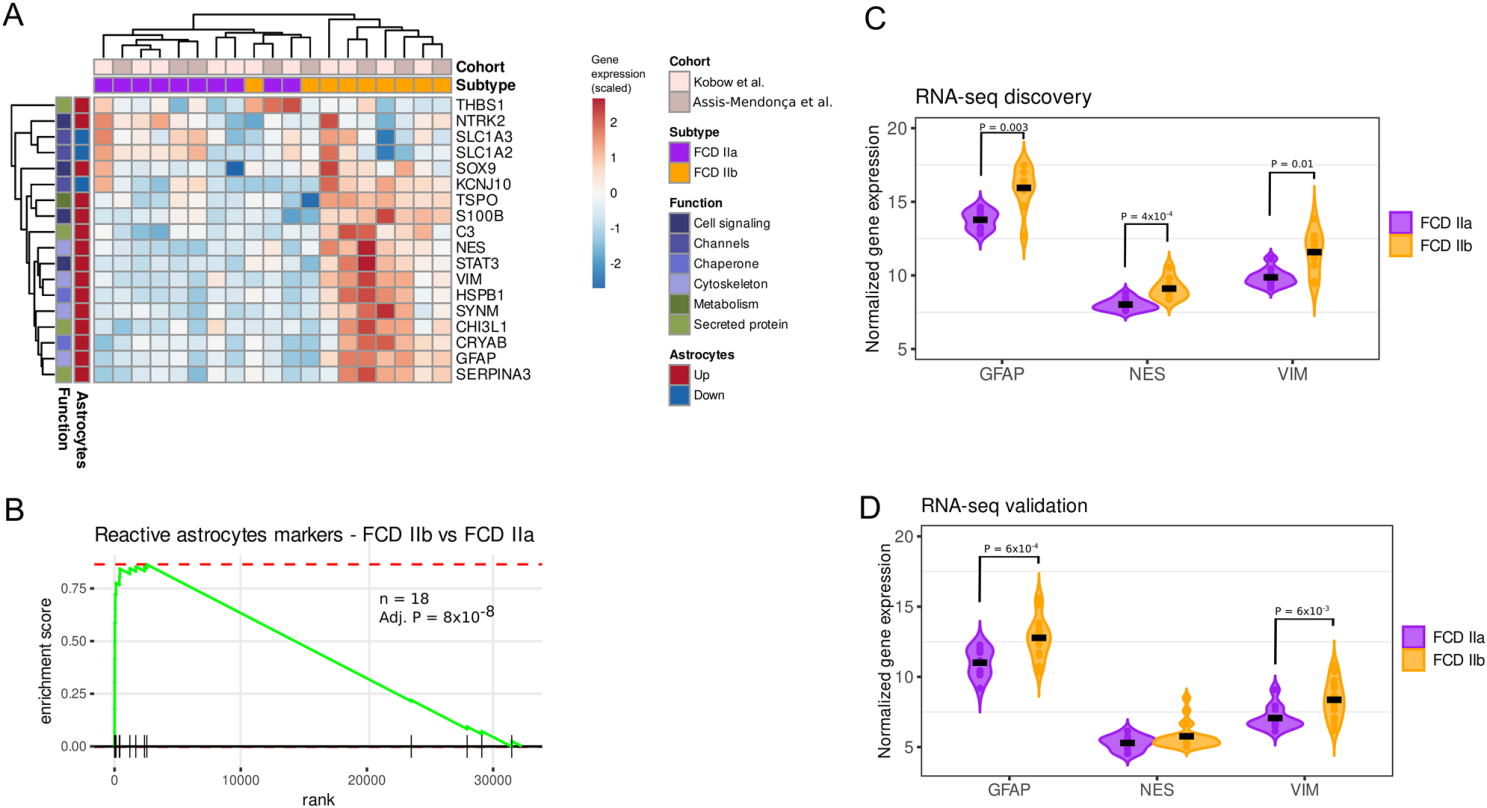
Reactive astrogliosis in FCD IIb lesions. (**A**) Heatmap expression of RA gene biomarkers in FCD IIb and IIa. Patient information (study and diagnosis) are indicated in the columns, and biomarker function and status (Up/Down-regulated in reactive astrocytes) are shown in the rows. (**B**) FGSEA plot of the RA signature (n = 18 genes, shown in A) based on expression FCD IIb. The *y*-axis indicates the enrichment score, and the *x*-axis represents all genes in the transcriptome ranked by log_2_ fold-change between FCD IIb and IIa. Genes in the RA signature are depicted by vertical lines along the *x*-axis. (**C**,**D**) Violin plots of GFAP, NES, and VIM gene expression in FCD IIa and IIb patients from RNA-seq data of (**C**) patient cohort 1 and (**D**) patient cohort 2. Significant changes between gene expression in FCD IIa and IIb were detected using the Wilcoxon rank-sum test (*P*-value indicated). Overlay dots represent gene expression in individual patients, and the black crossbar represents the median gene expression. Heatmap was created in R using the pheatmap package v.1.0.12 (https://cran.r-project.org/package=pheatmap).

Overall, we found that most astrogliosis biomarkers had increased expression in FCD IIb when compared to FCD IIa, and these genes were sufficient to group FCD II lesions by subtype (Fig. 3A). Using the FGSEA enrichment test^32^, we confirmed that the astrogliosis gene signature is highly expressed in the FCD IIb subtype (Fig. 3B). RNA-seq measurements of GFAP and VIM gene expression were significantly higher in FCD IIb in both patient cohorts, while NES was higher in the first cohort (Fig. 3C,D).

**Figure 4:**
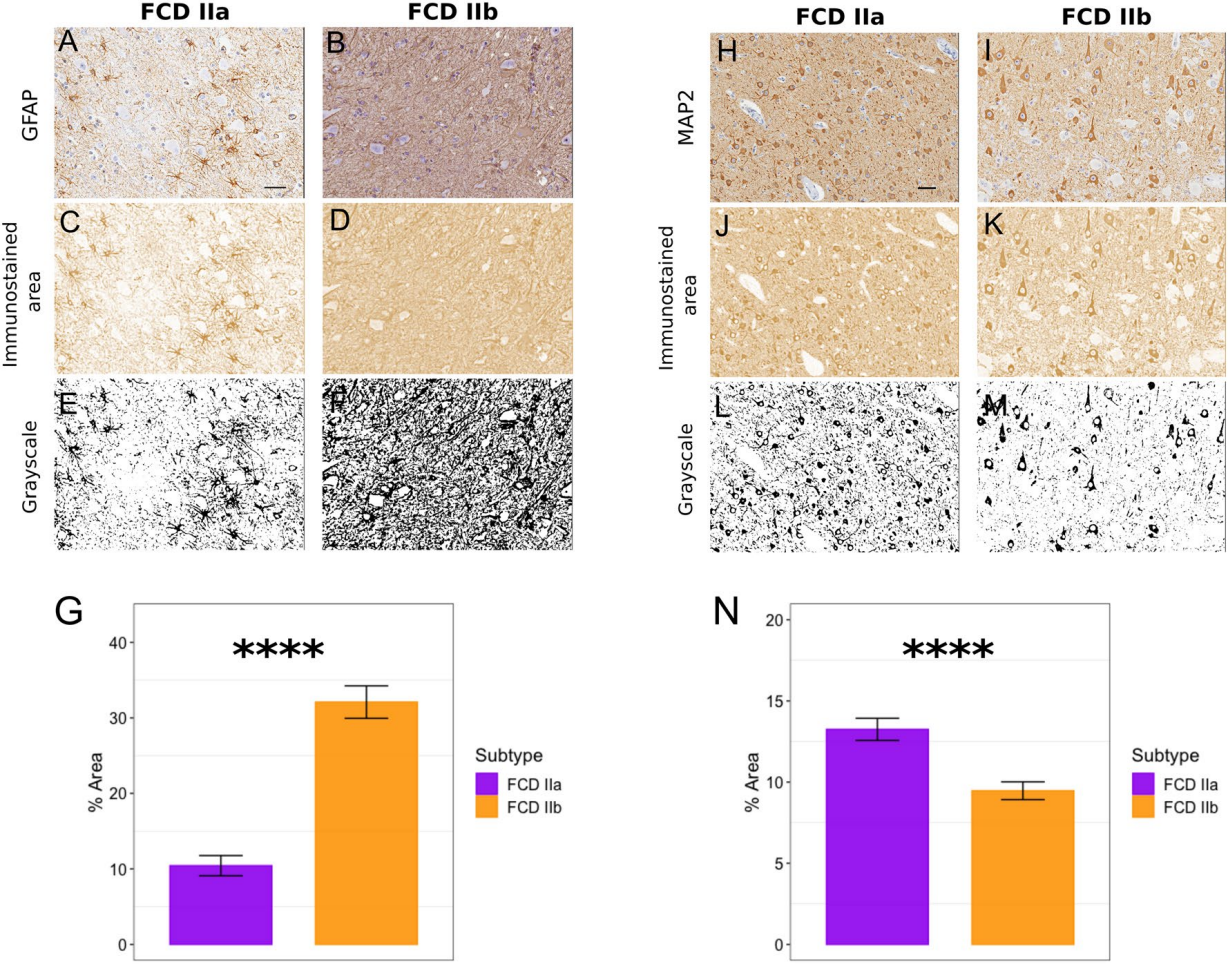
Immunostaining for GFAP and MAP2 proteins in FCD IIa and IIb tissues. Representative images of cortical immunostaining, DAB channel conversion, and grayscale conversion for (**A**–**F**) the astrocyte marker GFAP (glial fibrillary acidic protein), and (**H**–**K**) the neuron marker MAP2 (microtubule-associated protein 2). Barplots of GFAP (**G**) and MAP2 (**N**) expression levels in FCD IIa (n = 4) and IIb (n = 4) lesions. The mean expression was averaged from 10 randomly selected subfields in each lesion. The uncertainty bars represent 95% confidence intervals of the means. The image grayscale conversion of the positive immunostained area was used for marker quantification. Wilcoxon rank-sum test, ****p < 10^−4^. Scale bar: 50 μm.

To confirm our findings, we performed IHC staining for the astrocyte marker GFAP and neuronal marker MAP2 on cortical tissues from 8 patients with intractable epilepsy and a diagnosis of FCD IIa (n = 4) or IIb (n = 4, Fig. 4). To analyze GFAP and MAP2 immunoexpression, we randomly sampled ten fields per section in the neocortical region where the neuropathological findings characteristic of FCD type II, such as loss of cortical lamination and dysmorphic neurons—along with balloon cells in type IIb—were observed. The mean area of immunopositivity obtained from the DAB channel images was quantified for each sample and compared between FCD subtypes. The results showed that GFAP immunostaining was significantly higher in FCD IIb compared to FCD IIa specimens (Fig. 4A–G), indicating astrogliosis is more pronounced in FCD IIb lesions. In addition, MAP2 immunostaining levels were significantly lower in FCD IIb (Fig. 4H–N), suggesting that astrogliosis is also accompanied by neuronal loss in FCD IIb.

## Discussion

Cellular deconvolution is a powerful tool for analyzing transcriptomic and epigenetic data from complex tissues, where the contribution of different cell types to the overall gene expression profile is unknown or might be affected by disease. In this study, we leveraged this bioinformatic approach to comprehensively characterize the previously unknown cell type composition of FCD type II, a highly epileptogenic condition that affects mainly children and adolescents.

 Specifically, we applied cell-type deconvolution combined with single-cell signatures to estimate cell-type proportions in patients diagnosed with FCD type IIa and IIb. First, we conducted a systematic collection and processing of publicly available RNA-seq and methyl-seq datasets acquired from individuals diagnosed with FCD type II. Subsequently, we employed deconvolution techniques using various brain signatures derived from single-cell studies to attain an unbiased estimation of the cellular landscape within FCD type II lesions. We found that even though FCD type II lesions share common histological features, these FCD subtypes have remarkably distinct cellular profiles. Both transcriptome- and methylome-based cellular deconvolution analyses have shown that FCD IIb lesions are characterized by neuronal loss and astrocyte activation. In addition, our data showed that an astrogliosis gene signature is upregulated in FCD IIb. Further validation by IHC staining using MAP2 and GFAP markers confirmed these neuronal and glial changes in FCD IIb. Taken together, these findings indicate that neuronal loss and enhanced astrogliosis are characteristics of FCD IIb lesions that might be included as criteria for subtype classification.

Previous studies have reported evidence of astrogliosis in FCD IIb using standard immunohistochemical methods. Finardi et al.^33^ and Rossini et al.^34^ observed increased GFAP positivity in FCD IIb lesions compared to non-dysplastic adjacent tissue. Sousa et al.^35^ have also reported astrogliosis in FCD IIb tissue. Additionally, Miles et al.^36^ conducted a qualitative analysis and found that patients with FCD type II were more likely to exhibit diffuse astrogliosis compared to those with FCD type I.

The available data on neuronal loss in FCD IIb seem to present discrepant results. Nakagawa et al.^37^ found no differences in neuronal density between FCD IIb and postmortem controls. On the other hand, both Finardi et al.^33^ and Rossini et al.^34^ observed lower neuronal density in FCD IIb compared to non-lesion adjacent areas. Yet, these studies focused on FCD IIb specimens, without comparing FCD II subtypes.

In their study, Liang et al.^38^ found a reduction in the subpopulations of interneurons (INs), specifically parvalbumin (Pvalb) and somatostatin immunoreactive (Sst-IR) cells, when comparing FCD IIb and IIa. The decrease was specifically noted in the deep cortex region, while no changes were seen in the superficial cortex. In comparison, our deconvolution analysis of INs subpopulations (Fig. 2), estimated the population frequency across the entire lesion, encompassing all cortical layers. Consequently, as expected the deconvolution did not find significant changes in IN-Pvalb and IN-Sst frequencies between FCD II subtypes.

Our deconvolution also predicted an increase in microglia in the FCD IIb group compared to FCD IIa, particularly using the SR signature (Fig. 2). Boer et al.^39^ found that activated microglia (HLA-DR^+^ cells) have a higher frequency in FCD II than in controls, and were detected in clusters around blood vessels, dysplastic neurons, and balloon cells. Their study has not compared subtypes IIa and IIb, but our analysis suggests that this increase might be even more prevalent in FCD IIb lesions.

It is noteworthy that T2-weighted fluid-attenuated inversion recovery (FLAIR) magnetic resonance imaging of FCD IIb lesions reveals hyperintense signals in the cortical and subcortical regions, while FCD IIa lesions exhibit mild or absent hyperintense signals^40^. The discrepancy in FLAIR images between FCD II subtypes may be attributed to their cellular differences, even though further investigation is needed to explore this hypothesis.

Currently, the presence of balloon cells is the only histopathological feature used to distinguish between FCD type IIa and IIb lesions, and the status of glial cells is not taken into account in subtype classification. Based on our findings, measuring astrogliosis status by GFAP staining could be useful as an additional criterion for distinguishing between FCD IIb and FCD IIa. The analysis of GFAP status might be applied in conflicting cases where the clinical or neuroimaging findings suggest a type IIb lesion and the inspected tissue sample does not exhibit balloon cells^12^. Thus, as a current contribution of our work to neuropathological diagnosis, we propose to consider the pattern of GFAP immunolabelling when classifying IIa and IIb lesions.

Our computational analysis was designed to be as robust and comprehensive as possible. We searched public repositories to obtain all available RNA-seq and methylome data for FCD type II for which clinical annotation was available. We utilized both discovery and validation patient cohorts, containing transcriptomic data generated in three research centers. Finally, to rule out the effect of the underlying single-cell signature in cellular deconvolution, we reproduced the results using four independent single-cell signatures derived from single-cell studies of the cerebral cortex, which is the main site of tissue changes in FCD.

Furthermore, this study demonstrates the feasibility of deconvoluting available gene expression data, followed by experimental validation, as a viable approach to gaining insights into cellular populations associated with neurological conditions and discovering cellular markers in patient samples derived from complex tissues such as the brain. The identification of novel cellular markers through unbiased cell-type deconvolution can facilitate patient stratification and improve clinical diagnosis for neurological diseases.

In conclusion, our results indicate that the cellular environment in FCD IIb lesions is distinct from that of FCD IIa, with a higher level of astrogliosis in FCD IIb. This enhanced astrocyte response may contribute to increased neuroinflammation and the observed neuronal loss in FCD IIb. Further research is necessary to fully understand the role of astrocytes in FCD IIb and to investigate the potential therapeutic benefits of targeting astrocytes in the treatment of this condition.

### Supplementary Information

Supplementary Figures.

Supplementary Information 1.

Supplementary Information 2.

## Data Availability

RNA-seq analyzed in this study are available at the European Nucleotide Archive (accession code SRP188422) and at the Gene Expression Omnibus (accession code GSE213488) repositories. RNA-seq from Zimmer et al.^19^ was obtained from the authors. The source code to reproduce the cell-type deconvolution analyses is provided at https://github.com/icgalvao/DeconvolutionFCD.
