# Supplementary material for: Identifying cellular markers of focal cortical dysplasia type II with cell-type deconvolution and single-cell signatures
Source: Sci Rep. 2023 Aug 16;13:13321. doi: 10.1038/s41598-023-40240-3 (PMC10432381; doi:10.1038/s41598-023-40240-3)
Supplement: Supplementary file 1 — Supplementary Figures. [file 41598_2023_40240_MOESM1_ESM.docx]

**SUPPLEMENTARY FIGURES**


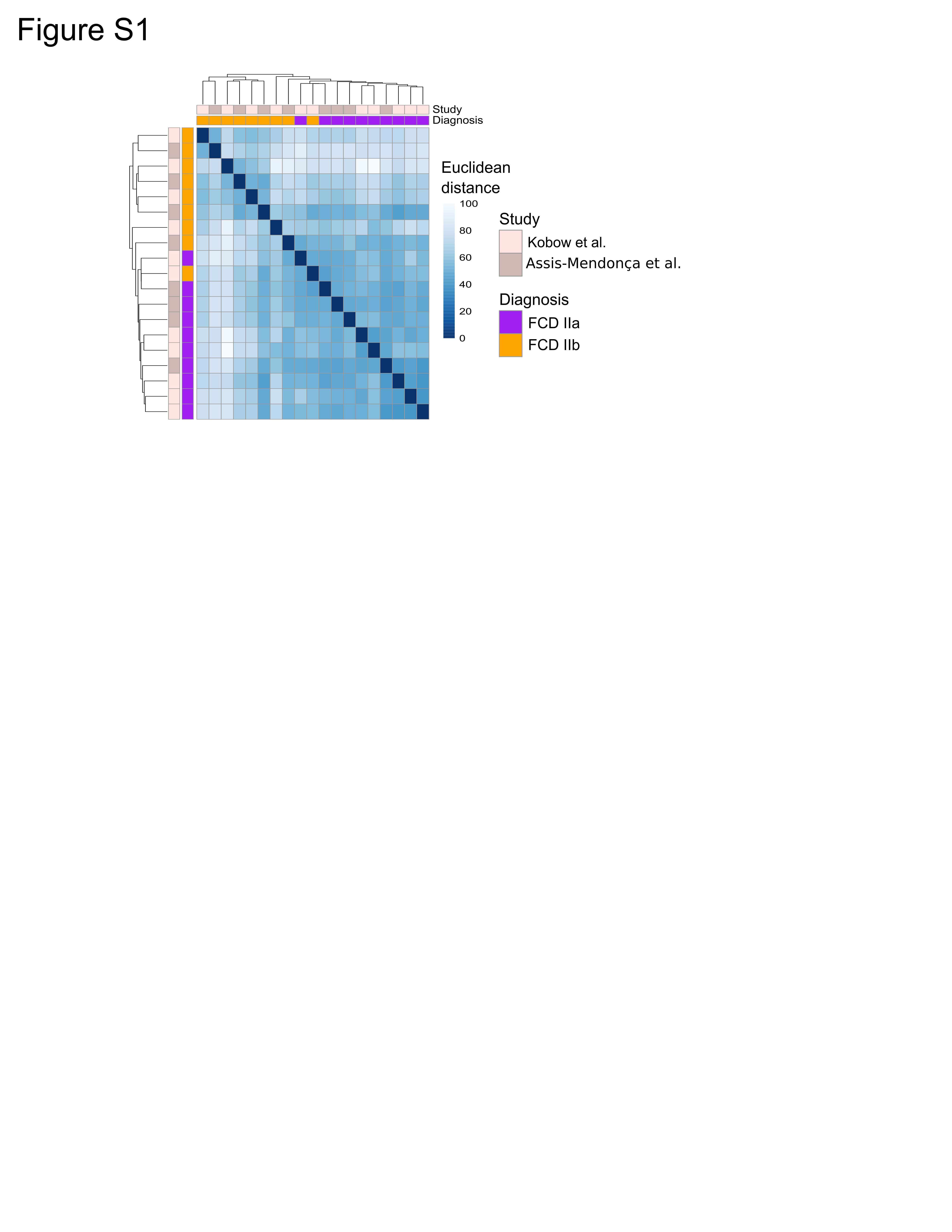
**Figure S1 - Hierarchical clustering of FCD type IIa and IIb lesions from patients in Dataset 1.** RNA-seq from two independent studies (Kobow et al and Assis-Mendonça et al) were integrated using a uniform pipeline, and gene levels were clustered using the Euclidean distance after batch correction (see Methods). Color in the heatmap indicates the Euclidean distance between samples. Study and diagnosis are indicated next to the heatmap. Heatmap was created in R using the pheatmap package v.1.0.12 (https://cran.r-project.org/package=pheatmap).


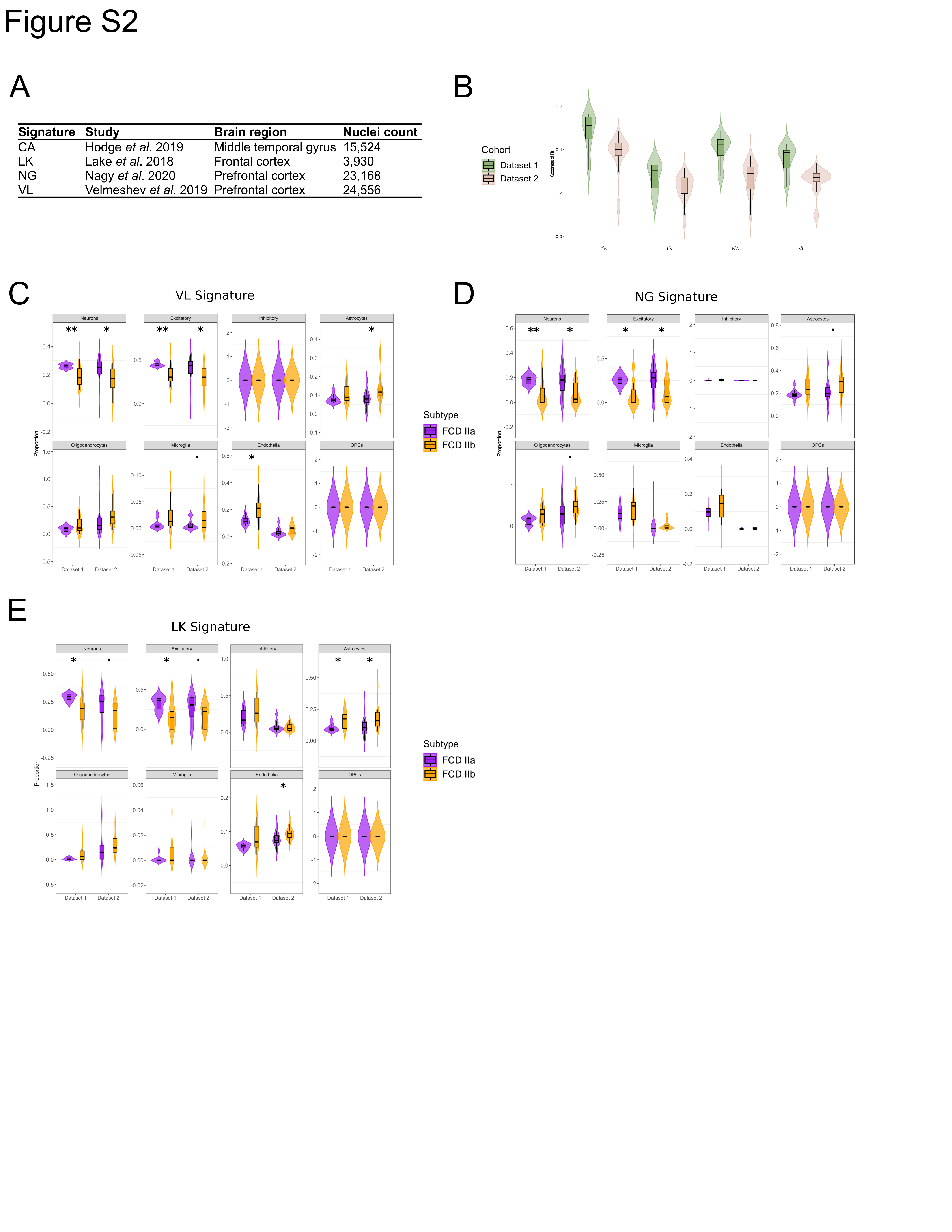


**Figure S2** - **Cellular deconvolution of major brain cell types in FCD IIa and IIb using multiple single-cell reference signatures.**

**(A)** Single-cell reference signatures used for cell-type deconvolution, derived from single-nuclei RNA-seq studies from regions of the cerebral cortex.

**(B)** Violin plots of the CIBERSORTx goodness-of-fit (i.e. Pearson correlation between the actual RNA-seq gene expression and reconstructed gene expression) for the reference signatures CA, VL, NG, and LK in Dataset 1 and Dataset 2. The width of the violin indicates sample density, with the top, middle, and bottom of the white boxplot marking the 75^th^, 50^th^, and 25^th^ percentiles, respectively.

**(C-E)** Violin plots of estimated cell type proportions in FCD IIa/IIb lesions in patients from Dataset 1 using the **(C)** VL signature, **(D)** NG signature and **(E)** LK signature. The *y*-axis indicates the absolute proportion (0 to 1) estimated by CIBERSORTx, while the *x*-axis indicates the patient dataset. The width of the violin indicates sample density, with the top, middle, and bottom of the white boxplot marking the 75^th^, 50^th^, and 25^th^ percentiles, respectively. Significant changes between cell-type estimates in FCD IIa/IIb lesions were detected using the Wilcox rank-sum test. ˙ P < 0,1, *P < 0.5, **P < 0.01, ***P < 10^-3^, **** P < 10^-4^.


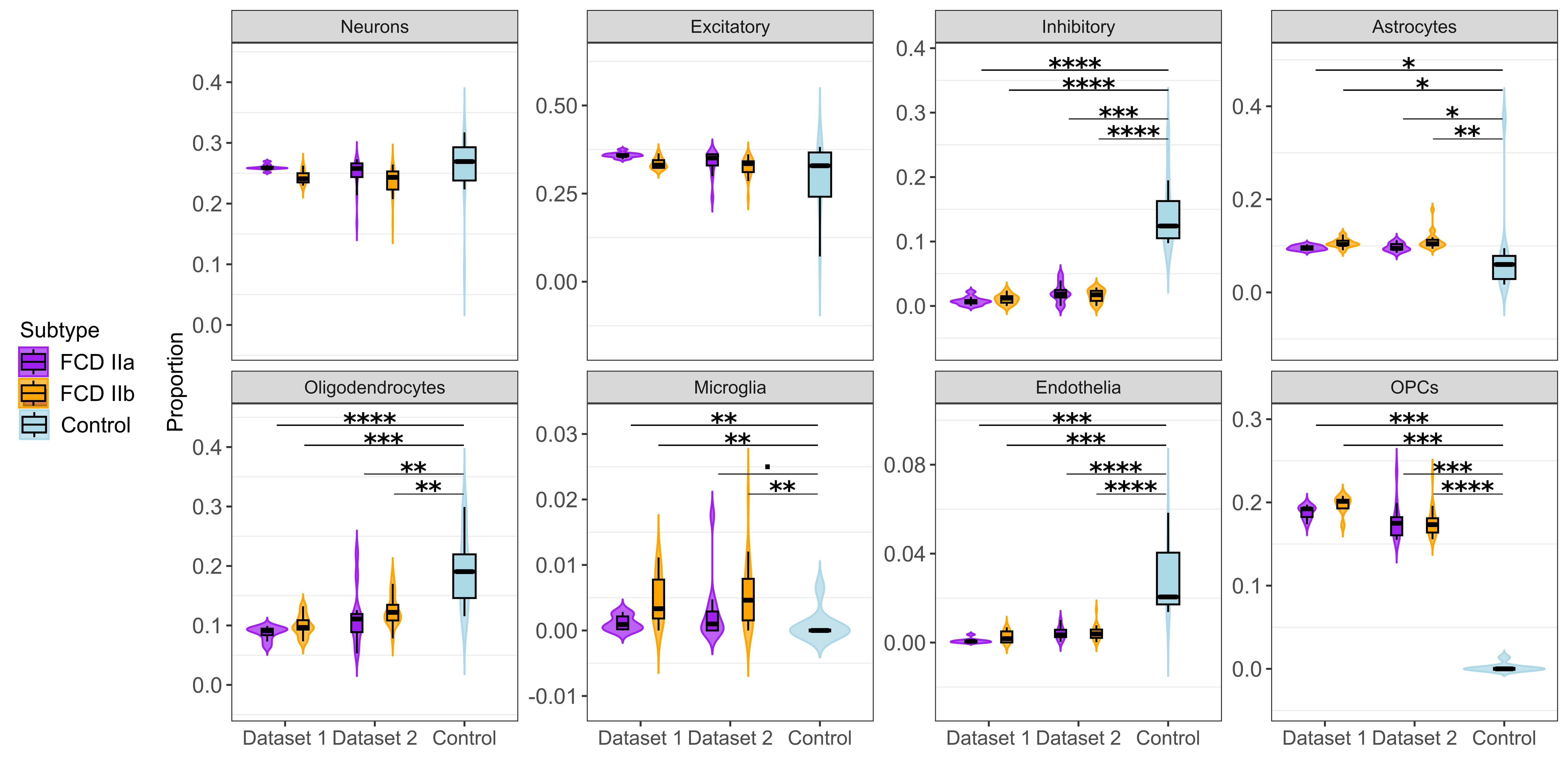


**Figure S3 – Comparison of cell-type frequencies between FCD type II subtypes and controls.** CIBERSORTx was applied to deconvolute RNA-seq of frontal cortex obtained from healthy age-matched controls from BrainSpan (n=8), using the CA signature. The *y*-axis indicates the absolute proportion (0 to 1) estimated by CIBERSORTx, while the *x*-axis indicates the patient dataset. The width of the violin indicates sample density, with the top, middle, and bottom of the white boxplot marking the 75^th^, 50^th^, and 25^th^ percentiles, respectively. Significant changes between cell-type estimates in FCD IIa/IIb lesions *vs* controls were detected using the Wilcox rank-sum test. For clarity, the significance is indicated only for the comparison between FCD subtypes *vs* controls. ˙ P < 0,1, *P < 0.5, **P < 0.01, ***P < 10^-3^, **** P < 10^-4^.


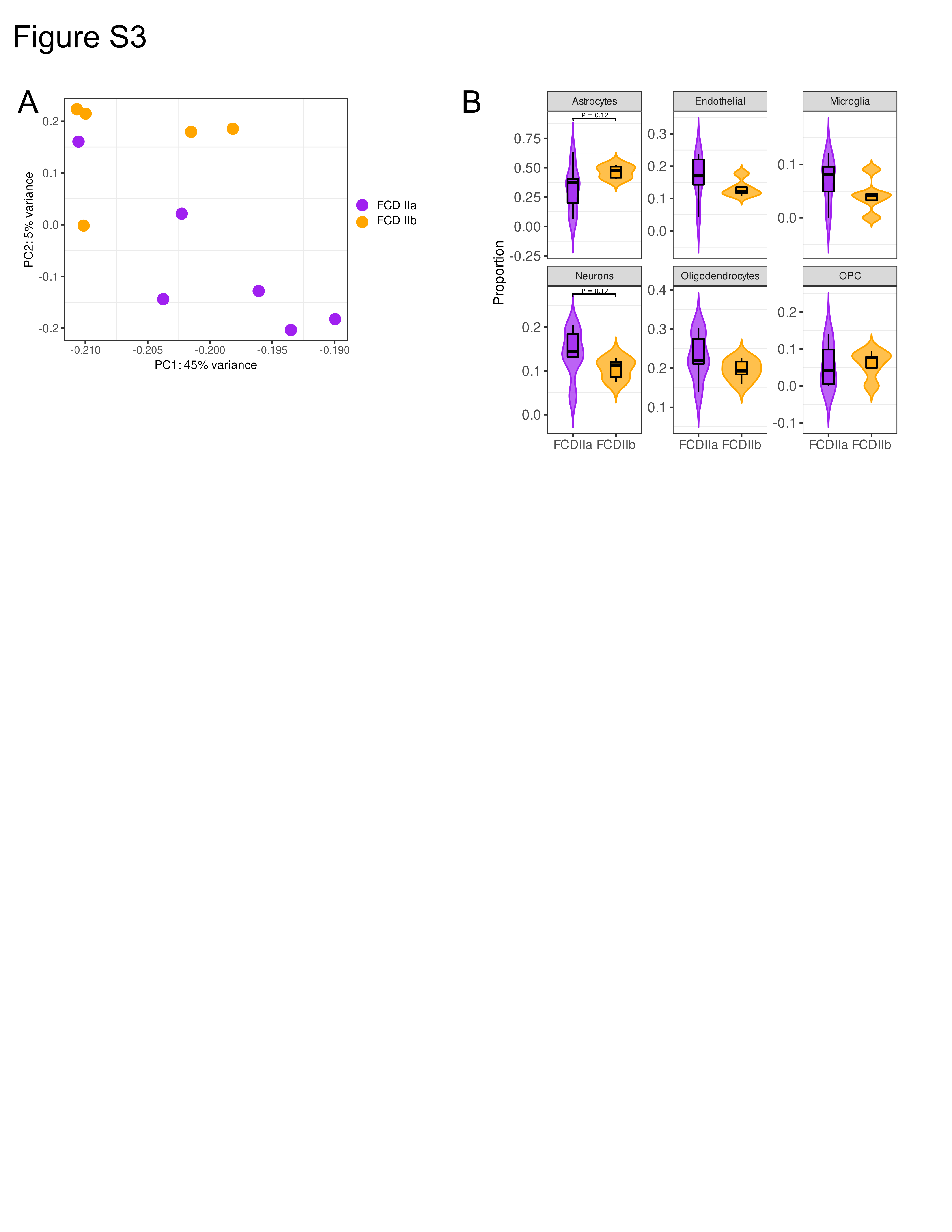
**Figure S4** - **Cellular deconvolution based on FCD methylomes.**

**(A)** Dot plots representation of FCD IIa/IIb methylomes after dimensionality reduction with principal component analysis (PCA). The *x*-axis and *y*-axis refer to the first and second PCA components, respectively.

**(B)** Violin plots of cell type proportions estimated by EpiScore in FCD IIa/IIb methylomes. The *y*-axis indicates the absolute proportion (0 to 1), while the *x*-axis indicates the disease subtype. The width of the violin indicates sample density, with the top, middle, and bottom of the white boxplot marking the 75^th^, 50^th^, and 25^th^ percentiles, respectively.
